# Does biofluorescence enhance visual signals in birds-of-paradise?

**DOI:** 10.1098/rsos.241905

**Published:** 2025-02-12

**Authors:** Rene P. Martin, Emily M. Carr, John S. Sparks

**Affiliations:** ^1^ School of Natural Resources, University of Nebraska-Lincoln, Lincoln, Nebraska, USA; ^2^ American Museum of Natural History, New York, NY, USA; ^3^ Richard Gilder Graduate School, New York, NY, USA

**Keywords:** biofluorescence, Paradisaeidae, sexual selection

## Abstract

Visual signals are important for mediating numerous behaviours in organisms. Frequently, brightly coloured feathers are used in signalling during reproductive behaviours in birds. Furthermore, an increasing number of studies have documented the additional use of biofluorescence as a visual cue. We investigate the presence of fluorescence in all 45 species of birds-of-paradise (Paradisaeidae), a group where males exhibit elaborate feather morphology, coloration, and mating displays. We show that all core birds-of-paradise are biofluorescent (37 species representing 14 of 17 genera); all genera except *Lycocorax*, *Manucodia* and *Phonygammus*, which comprise the sister group to the core birds-of-paradise. In males, biofluorescence occurs on plumage and skin used in reproductive displays. Biofluorescent regions vary among species but include the inner mouth and bill, as well as feathers on the head, neck, belly and plumes. In females, biofluorescence is usually restricted to plumage on the chest and belly. Emitted biofluorescent wavelengths are green and green-yellow, with emission peaks around 520 and 560 nm. Using an established framework of criteria for determining the functional role of biofluorescence in communication, our results provide evidence that within core birds-of-paradise, males likely utilize biofluorescence to enhance visual cues used during male hierarchy and mating displays.

## 1. Introduction

In many animals, vision and its associated visual cues are an important sensory system for mediating a variety of behaviours [1–5]. Eye morphology is similar among vertebrates but has differentiated and specialized in aspects of acuity, sensitivity and image resolution among species [6,7]. Birds are highly visual animals that are believed to use vision as their primary sense [6,8]. This exceptional visual acuity and sensitivity allows birds to fly with accuracy and precision. Although visual acuity varies greatly, some of the highest reported acuities of vertebrates are found in avian lineages [4,8]. Superior vision also helps birds discern visual cues associated with other important behaviours like finding food [9,10] and evading predators [11]. Additionally, reproduction and copulatory success are extremely important, and the visual cues birds use that are associated with reproductive behaviours are some of the most exorbitant in the vertebrate world, especially those used by males [12,13].

Males of many bird species are well known for their bright feathers [14–16] and intricate mating rituals [16,17]. Males often utilize their dazzling plumage during these courtship displays, while observant females are believed to be assessing male quality [17]. Visual cues associated with reproduction in birds have been the topic of numerous investigations into avian evolution, sexual selection [18–20] and in determining the type of information females derive during male courtship displays [21,22]. Understanding the importance of the visual cues utilized in these displays is intimately tied to understanding what the bird’s eye can discern and perceive.

Birds exhibit an incredibly complex cone photoreceptor system, comprising five different types of cone cells [23]. Their four single-cone cell types permit tetrachromatic colour vision. As a result, not only can they detect cues in the spectrum visible to the human eye, but many species can also visualize spectra within the ultraviolet (UV) range [24–27]. Birds also possess oil droplets on the cones in their eyes, which filter light and narrow the spectral sensitivity of each cone type, allowing them to better distinguish between colours [25,28]. Additionally, UV sensitivity varies greatly across birds, and some species may even possess additional intraocular filters, which reduce the amount of UV wavelengths that make it to the retina [5]. These filters are believed to help mitigate UV damage or reduce chromatic aberration [5]. In other vertebrates that possess intraocular filters, such as fishes [29], pigmented filters are believed to help individuals visualize another type of light signal, biofluorescence [30].

Biofluorescence occurs when an organism absorbs high-energy wavelengths of light (UV, violet and blue) and re-emits them at lower-energy wavelengths (greens, yellows, oranges and reds). Recent work has focused on examining the presence of biofluorescence and its use as a visual cue in organisms across the tree of life [31–33]. Despite there being over 10 000 described avian species, with numerous studies that have documented their bright plumage and excellent vision, only a handful have investigated the presence of biofluorescence. Within birds, auks, puffins and penguins [34–38], nocturnal owls and nightjars [39,40], parrots [26,41–44] and bustards [45] are the only lineages with published accounts of biofluorescence. This is surprising considering the amount of research devoted to assessing vision and the use of visual cues in birds, especially those associated with reproduction and mating. Of the studies listed above, only those focusing on parrots [26,43,44] and auks and puffins [34–37] have suggested a potential role of biofluorescence as a visual cue in courtship and copulation. In parrots, the budgerigar (*Melopsittacus undulatus*) may use biofluorescent plumage in courtship displays as a signal or to enhance contrast against other highly visible plumage that reflects UV light [43,44]. Among numerous other parrot species, fluorescent feathers are significantly associated with plumages used in courtship displays and are often accompanied by UV-reflective plumage [26]. In multiple species of auks and puffins, biofluorescence occurs from the cere, bill and bill plates, and one species (*Fratercula corniculate*) exhibits sexual dimorphism in the size of biofluorescent patches [34–37]. Conspicuous bill structures in these groups are thought to play a role in reproductive signalling, and additional biofluorescent emissions may aid as a visual cue if receptive parties can see them.

As additional studies continue to reveal how phylogenetically widespread biofluorescence is across the tree of life, one major question is whether biofluorescent emissions are functionally significant to the organisms that emit them. There are numerous instances of organisms with fluorophores in areas of the body, but in which biofluorescence is a by-product of other biological phenomena (e.g. fluorescence of human teeth). Marshall and Johnsen [46] and Nicolaï *et al*. [47] suggest using a framework for attributing function to biofluorescence and offer conditions to determine whether biofluorescent emissions should be considered visually significant to the individuals expressing them: (i) their biofluorescent pigments (fluorophores) are able to take advantage of excitation light wavelengths in the habitat the individuals live in; (ii) their fluorescent signals are viewed next to or against contrasting backgrounds; (iii) they are located in areas of the body that are involved in signalling; and (iv) that signal-receivers have optimal spectral sensitivities to the emitted wavelengths. In this study, we aim to understand whether biofluorescence is present and, if so, whether it could potentially function as visual signals in the birds-of-paradise (Paradisaeidae).

The 45 paradisaeid species occur across eastern Australia, Indonesia and New Guinea [48] and are recovered as the sister group to the monotypic New Guinea endemic, Ifritidae [19]. Male birds-of-paradise are well-known for their bright feather coloration, head, tail and plume ornamentations, and their numerous courtship signals and displays [49]. Paradisaeid courtship rituals and their extremely sexually dimorphic and elaborate plumages have been the focus of numerous sexual selection studies [22,50,51]. Given the variation in the extravagant plumage of male birds-of-paradise and the presence of biofluorescent plumage in another group of colourful birds (parrots), we hypothesized male birds-of-paradise may be using biofluorescence to enhance the visual signals they use in reproductive displays. Unfortunately, there are extreme difficulties gaining access to live birds-of-paradise to document and assess the use of biofluorescence during courtship displays. Observing and recording biofluorescence often requires the use of bright high-energy lighting and filters over camera lenses while imaging in a darkened room or setting, a complex task. Instead, we studied the extensive collection of bird-of-paradise skins at the American Museum of Natural History; natural history museums house an abundance of specimens collected through countless expeditions across centuries. Using this extensive collection and biofluorescent imaging and recording techniques, we aim to answer the following questions: (i) Are there species within Paradisaeidae that are biofluorescent and, if so, are the fluorescent regions of plumage and tissue sexually dimorphic? (ii) Excitation maximum (intensity) of specific fluorophores can vary based on excitation wavelength used (but not re-emission range). So, testing under both blue and UV excitation light, what are the wavelengths of biofluorescent light re-emitted and is there variation in wavelengths within an individual (different fluorophores) or among species? (iii) Does any biofluorescent patterning occur in association with body parts and feathers that are used in courtship/arena displays? (iv) Phylogenetic patterns in traits within a lineage can inform our understanding of their evolutionary history. To this end, is there any evidence of a phylogenetic pattern in biofluorescence across Paradisaeidae? (v) If biofluorescence is present, are the criteria outlined above for functional use as a visual signal satisfied?

## 2. Material and methods

### 2.1. Specimens

Previously prepared bird-of-paradise specimen skins were used in this study from the ornithology collection at the American Museum of Natural History (AMNH) in New York, which houses all currently described species of Paradisaeidae. Initial screening for the presence of biofluorescence was performed on both adult males and females for all 45 described species (except for *Parotia berlepschi*, where only males were analysed; females are not present in the collection) via excitation using SOLA NightSEA blue hand-held lights in a darkened collection room and while wearing longpass filter goggles to block excitation wavelengths. Species exhibiting potential biofluorescence were taken into a fully dark room for imaging and emission spectra analysis. To better understand the possible extent and evolution of biofluorescence across the group, additional specimens from the most closely related monotypic sister family Ifritidae (*Ifrita kowaldi*) were also analysed. A minimum list of the 110 specimens analysed can be found in electronic supplementary material, table S1. Additional specimens for each species were assessed for biofluorescence while working through the American Museum of Natural History’s collection of Paradisaeidae, which includes over 3500 specimen skins.

### 2.2. Spectrometry

While in a dark room, blue excitation light (royal blue; excitation of 440−460 nm) from a NightSEA stereo microscope fluorescence adapter was directed on specimens from variable distances, usually between 15 and 30 cm away, determined by intensity value graphical bounds (see below). While the specimens were illuminated by blue excitation light, fluorescence emission spectral readings were taken using an Ocean Optics USB2000+Fibre Optic Spectrometer (Dunedin, FL) equipped with a hand-held fibre optic probe (Ocean Optics ZFQ−12135). The spectrometer was connected to a computer with the fibre optic probe situated over specific anatomical parts of individuals and fluorescent spectral readings were recorded using the OceanView software v. 1.6.7. Appropriate distances and angles in which the probe was held were determined by the live spectral graph available in the OceanView software. Fluorescent emissions that were too bright resulted in intensity levels exceeding the graphical bounds; thus, the probe was moved away from the specimen until the intensity curves and peaks on the live spectral graph were visible and with intensity values not exceeding graphical parameters. Measurements were repeated multiple times for each specimen (and often for multiple specimens of a single species) to ensure accuracy of the fluorescent emission peak location and shape. Most fluorescent patches on species were large compared with the probe size, so readings only reflect emissions from these patches and not surrounding areas. Additionally, we record readings of the areas adjacent to fluorescent patches, which were usually dark, non-fluorescent plumage or structurally coloured plumage that reflects back the blue excitation light. Readings were not averaged, as variation in probe distance results in relative intensity readings. We report biofluorescent emission peaks, which are the range in which the wavelengths correspond with the highest intensity values.

To determine if UV light also excited biofluorescence in members of Paradisaeidae, excitation via use of a UV light (Analytik Jena US UVP 3UV lamp) was performed on an additional five species, which were previously found to be biofluorescent under blue light. Once biofluorescence under UV excitation was determined and recorded on these specimens, most imaging and spectral readings were performed under royal blue excitation to reduce the unnecessary risk associated with UV exposure.

### 2.3. Biofluorescence imaging and analysis

Biofluorescent specimens were imaged using a Nikon D800 digital camera outfitted with an AF-S Micro Nikkor 105 mm f/2.8 g ED lens. Blue excitation light was directed at specimens using the NightSEA Stereo Microscope Fluorescence Adapter, SOLA NightSEA hand-held lights and Nikon Speedlight SB-910 flashes covered with blue interference bandpass excitation filters (Omega Optical, Inc., Brattleboro, VT; Semrock, Inc., Rochester, NY). To filter out the blue excitation light and restrict imaging to wavelengths longer than approximately 500 nm, a 514 nm longpass (Semrock Inc., Rochester, NY) filter was attached to the camera lens prior to imaging. Biofluorescent images of specimens were altered slightly for publication as follows. The cotton used in the preparation of preserved specimens contains fluorophores and fluoresced in our images. Cotton is visible in specimen eye sockets and was edited out of images to better visualize biofluorescent patterns. Additionally, fluorescent specimen tags were also darkened on images for a similar purpose. Biofluorescent and white-light images were also slightly edited to remove dust particles and aberrant background debris.

Spectral intensity readings were graphed using R [52] via R studio [53] and the function ‘*ggplot*’ in the package ggplot2 [54]. In order to assess phylogenetic patterns in the presence or absence of biofluorescence in the birds-of-paradise, we recreated the phylogeny from Ligon *et al*. [16], which incorporates information from Iristedt *et al*. [19,55] by using the function ‘*read.tree*’ in the R package ape [56] adding *Ifrita kowaldi* as the root. We performed stochastic character mapping of biofluorescence on the tree using the function ‘*fitMk*’ from the package phytools [57]. We assessed the evolution of this trait across the birds-of-paradise using an equal-rates model and a symmetrical model.

## 3. Results

### 3.1. Patterns of biofluorescence in birds-of-paradise

**Figure 1.**
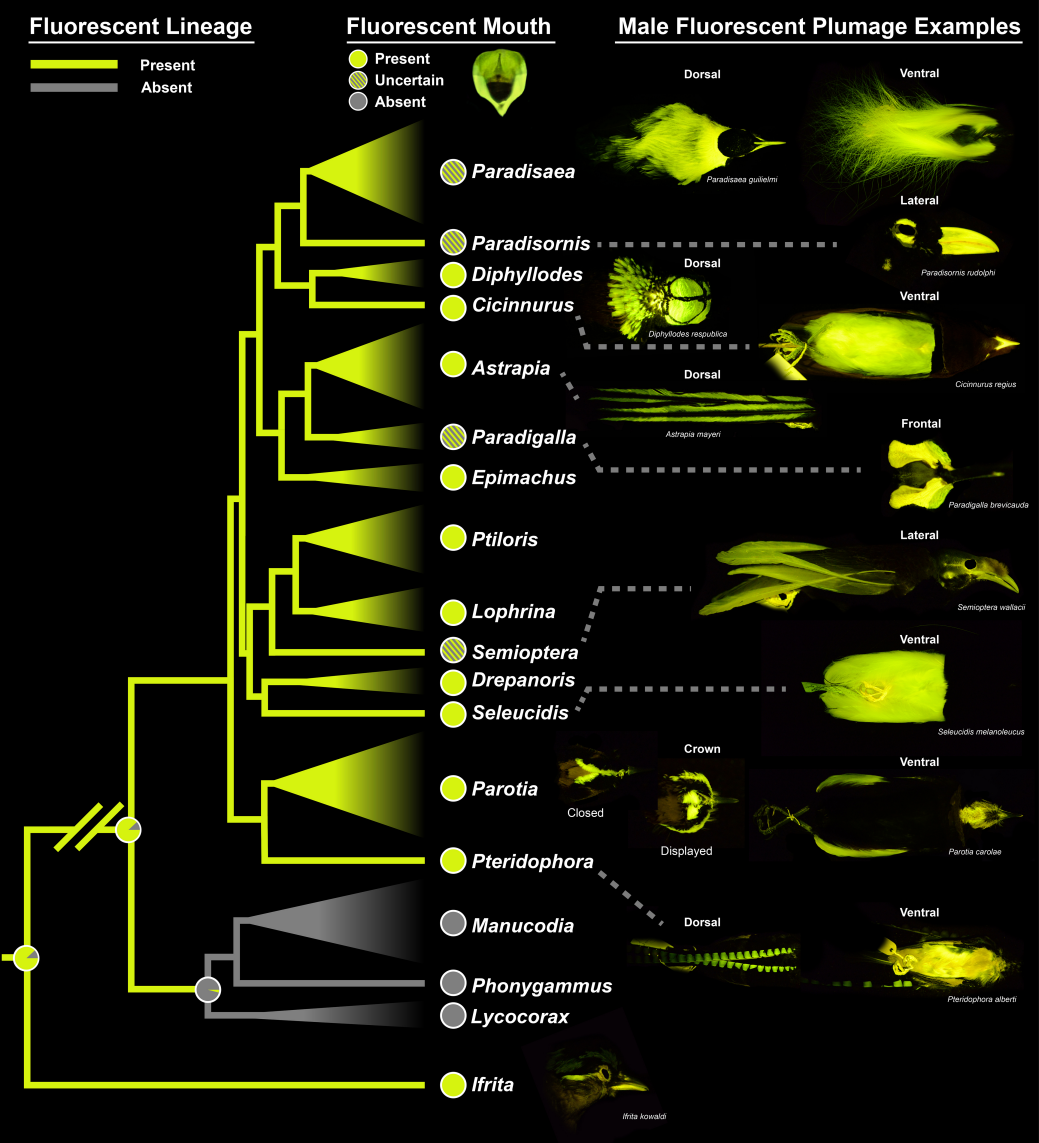
Stochastic character mapping of biofluorescence presence and absence on the bird-of-paradise phylogeny from Ligon *et al*. [16] with the addition of *Ifrita kowaldi* as the outgroup. Branches are coloured green if the ancestral character state for that lineage was 100% likely to be biofluorescent, otherwise likelihood pie charts from the symmetrical model are shown at nodes. Imaged male specimens include *Paradisaea guilielmi* (AMNH 303107), *Paradisornis rudolphi* (AMNH 679027), *Diphyllodes respublica* (AMNH 292440), *Cicinnurus regius* (AMNH 303137), *Astrapia mayeri* (AMNH 705537), *Paradigalla brevicauda* (AMNH 705505), *Semioptera wallaci* (AMNH 467439), *Seleucidis melanoleucus* (AMNH 677789), *Parotia carolae* (AMNH 302972; AMNH 678134), *Pteridophora alberti* (AMNH 678683) and *Ifrita kowaldi* (AMNH 200213). Biofluorescent mouth (image at centre top) created to illustrate what it might look like in life.

**Figure 2.**
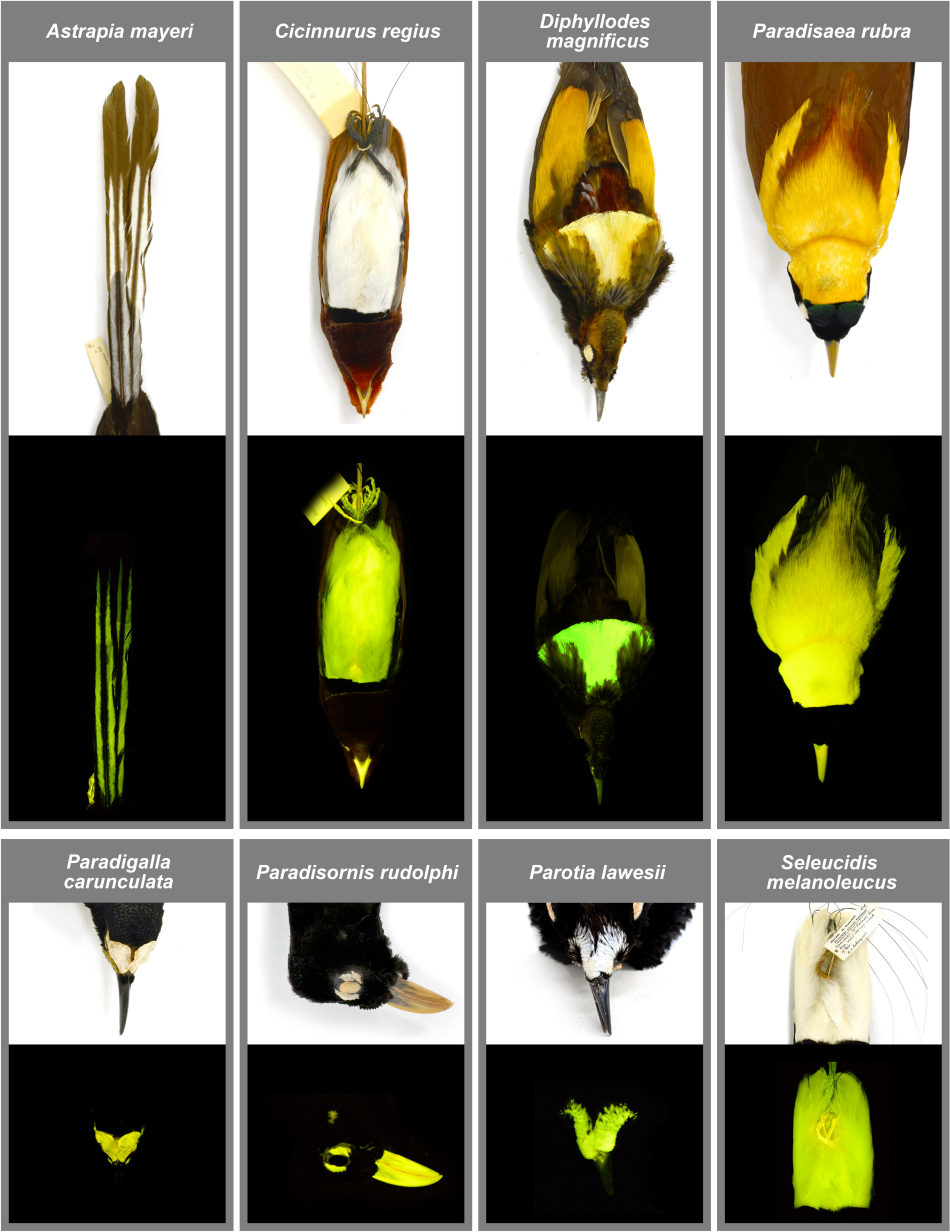
Examples of eight different male bird-of-paradise species imaged under white light (above) and also showing biofluorescent regions (below). *Astrapia mayeri* (AMNH 705537), *Cicinnurus regius*, (AMNH 303137), *Diphyllodes magnificus* (AMNH 303088), *Paradisaea rubra* (AMNH 300993), *Paradigalla carunculata* (AMNH 678344), *Paradisornis rudolphi* (AMNH 679027), *Parotia lawesii* (AMNH 330458) and *Seleucidis melanoleucus* (AMNH 677789).

**Figure 3.**
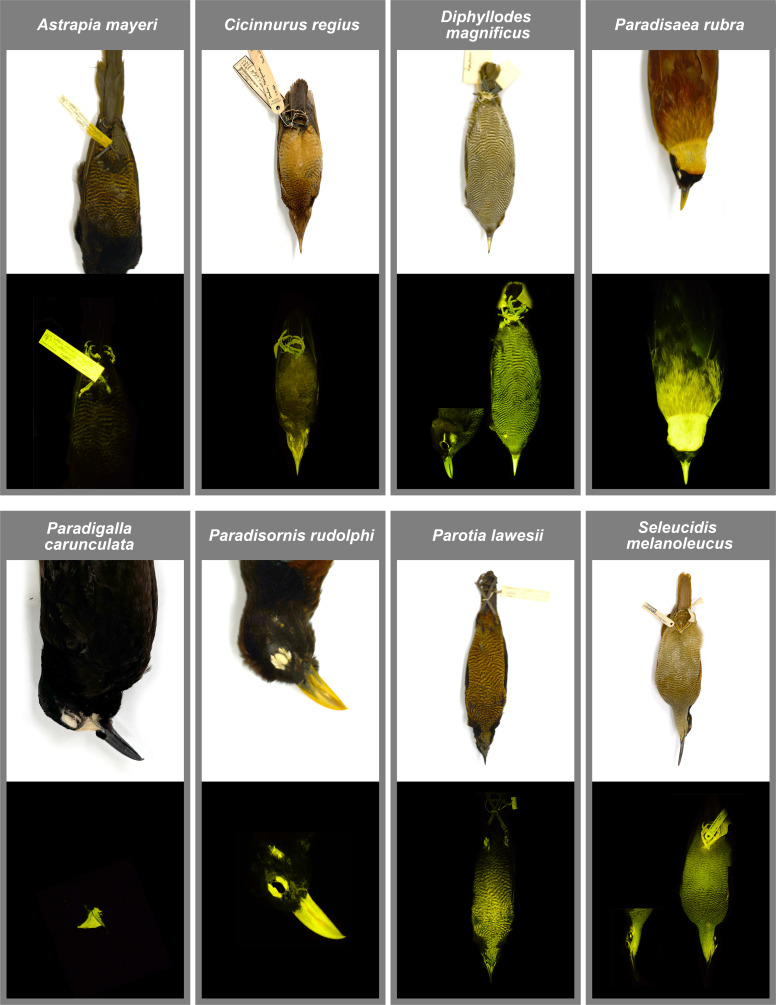
Examples of eight different female bird-of-paradise specimen counterparts to the species of males shown in figure 1 imaged under white light (above) and also showing biofluorescent regions (below). *Astrapia mayeri* (AMNH 705577), *Cicinnurus regius*, (AMNH 678539), *Diphyllodes magnificus* (AMNH 678384), *Paradisaea rubra* (AMNH 301004), *Paradigalla carunculata* (AMNH 294611), *Paradisornis rudolphi* (AMNH 679039), *Parotia lawesii* (AMNH 420983) and *Seleucidis melanoleucus* (AMNH 677798).

**Figure 4.**
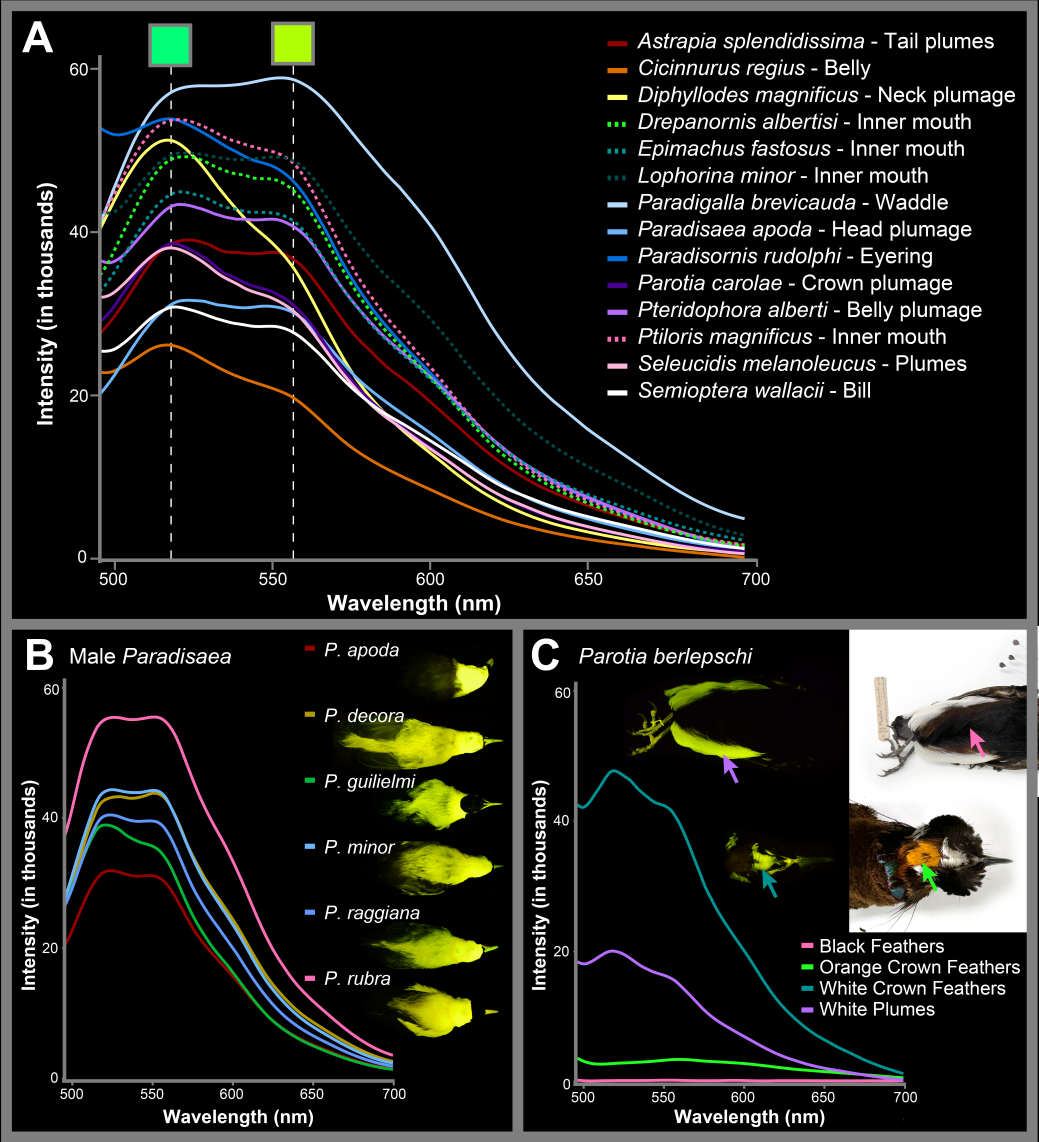
Spectra readings of biofluorescent patches in males (A) among genera (dotted lines denote inner-mouth spectra readings), (B) among species within the genus *Paradisaea* (*P. apoda*, AMNH 678750; *P. decora*, AMNH 784693; *P. guilielmi*, AMNH 303107; *P. minor*, AMNH 9795; *P. raggiana*, AMNH 268993; *P. rubra*, AMNH 300993) and (C) from different patches on an individual of *Parotia berlepschi* (AMNH 678171). Note: absolute intensity values in this study are only comparable within the same spectral reading (line) on the graph and are not comparable among different spectral readings.

We detected biofluorescence in 37 of the 45 bird-of-paradise species and 14 of the 17 genera (all genera except *Lycocorax*, *Manucodia* and *Phonygammus*; figure 1; table 1). These 14 biofluorescent genera represent all core birds-of-paradise. If biofluorescence occurred on the plumage, plumage patterns were sexually dimorphic in all assessed species except *Paradisornis* (figures 1–3; electronic supplementary material, figure S1). In males, we detected biofluorescence on the plumage of 19 species, in areas of the body associated with courtship rituals (figure 2). These biofluorescent plumage patches are generally similar in location among species within a genus (figure 4B). Biofluorescent plumage commonly occurs on the head, neck/nape and plumes in species of *Diphyllodes*, *Paradisaea*, *Parotia*, *Seleucidis* and *Semioptera* (figure 2, electronic supplementary material, figure S1). Less-common locations for biofluorescent plumage in males can be found on the breast and belly in male *Cicinnurus*, *Pteridophora* and *Seleucidis* species (figure 2), on the elongate tail feathers in male *Astrapia*, and on the feather rings around the eyes in male *Paradisornis* and *Ptiloris*. Biofluorescence also emanates from the feet of many, but not all, species (figure 2; electronic supplementary materials). The males of an additional two species (genus *Paradigalla*) have non-fluorescent plumage but possess large biofluorescent, non-sexually dimorphic face waddles (figure 2).

**Table 1.** All bird-of-paradise species indicating the presence (X) or absence (-) of biofluorescence. UV spectral readings were also taken for starred (*) species names. Spectral readings of the inner mouth were taken in X^m^ species. The inner mouths of some species were difficult to assess on museum specimens (see §2), and thus biofluorescence was either coded as likely or unknown (?).

genus	species	male plumage or waddle biofluorescence	male mouth / throat biofluorescence	female biofluorescence
*Astrapia*	*mayeri*	X	X	X
*Astrapia*	*nigra*	—	X	X
*Astrapia*	*rothschildi*	—	X	X
*Astrapia*	*splendidissima*	X	X	X
*Astrapia*	*stephaniae*	—	X	X
*Cicinnurus*	regius*	X	X	X
*Diphyllodes*	magnificus*	X	X	X
*Diphyllodes*	respublica*	X	X	X
*Drepanornis*	*albertisi*	—	X^m^	X
*Drepanornis*	*bruijnii*	—	X	X
*Epimachus*	*fastosus*	—	X^m^	X
*Epimachus*	*meyeri*	—	X^m^	X
*Lophorina*	*minor*	—	X^m^	X
*Lophorina*	*niedda*	—	X^m^	X
*Lophorina*	*superba*	—	X^m^	X
*Lycocorax*	*obiensis*	—	—	—
*Lycocorax*	*pyrrhopterus*	—	—	—
*Manucodia*	*alter*	—	—	—
*Manucodia*	*ater*	—	—	—
*Manucodia*	*chalybatus*	—	—	—
*Manucodia*	*comrii*	—	—	—
*Manucodia*	*jobiensis*	—	—	—
*Paradigalla*	*brevicauda*	X	?	X
*Paradigalla*	*carnculata*	X	?	X
*Paradisaea*	*apoda*	X	ikely	X
*Paradisaea*	*decora*	X	likely	X
*Paradisaea*	guilielmi*	X	likely	X
*Paradisaea*	*minor*	X	likely	X
*Paradisaea*	*raggiana*	X	likely	X
*Paradisaea*	*rubra*	X	likely	X
*Paradisornis*	*rudolphi*	X	likely	X
*Parotia*	*berlepschi*	X	X	N/A
*Parotia*	carolae*	X	X	X
*Parotia*	*helenae*	—	X	X
*Parotia*	*lawesii*	X	X	X
*Parotia*	*sefilata*	X	X	X
*Parotia*	*wahnesi*	—	X	X
*Phonygammus*	*keraudrenii*	—	—	—
*Pteridophora*	*alberti*	X	X	X
*Ptiloris*	*intercedens*	—	X^m^	X
*Ptiloris*	*magnificus*	—	X	X
*Ptiloris*	*paradiseus*	—	X^m^	X
*Ptiloris*	*victoriae*	—	X^m^	X
*Seleucides*	*melanoleucus*	X	X	X
*Semioptera*	*wallacii*	X	moderately	X

All males that possess biofluorescent plumage or waddles also likely possess biofluorescent regions of the inner mouth and throat (figure 1; table 1). An additional 16 species, including all species in *Drepanornis*, *Epimachus*, *Lophorina* and *Ptiloris* that do not possess biofluorescent plumage or waddles, and additional non-fluorescent plumaged species in *Astrapia* and *Parotia,* also exhibit fluorescent regions of the inner mouth and throat. Although we confirmed the presence of biofluorescence of the inner mouth via spectral readings of some species (table 1, starred; figure 4A dashed lines) and through visual observation in most others, specimen skins are usually prepared with fully closed bills which are often filled with cotton or other materials. This presents a barrier in obtaining spectral readings from the inner mouth of most specimens. Although many bird-of-paradise species possess dark-coloured non-fluorescent bills (e.g. *Parotia lawesii*, *P. wahnesi*), we encountered further difficulty in assessing the presence of a fluorescent inner mouth in some species with biofluorescent bills (e.g. *Paradisaea*, *Semioptera*) because it was difficult to determine if only the bill was fluorescing or if the skin of the inner mouth was also fluorescent (electronic supplementary material, figure S2A).

In females, biofluorescence occurs in 36 (likely 37) species in the same 14 genera where it is found in males (table 1) and is present in all core birds-of-paradise. Similar to males, biofluorescence does not occur in females of *Lycocorax*, *Manucodia* and *Phonygammus*. When biofluorescence occurs in females in *Cicinnurus*, *Diphyllodes*, *Epimachus*, *Lophorina*, *Parotia*, *Pteridophora*, *Ptiloris* and *Seleucidis*, it is located most frequently on the patterned and mottled feathers of the chest and belly and is also often present in feathers that occur as an eye stripe on the side of the head (figure 3). Only females of species in *Diphyllodes*, *Paradisaea*, *Paradisornis* and *Semioptera* possess somewhat similar biofluorescent plumage patches to males (figures 2 and 3). One female in *Paradisaea*, *P. minor*, possesses a bright biofluorescent belly. Females in *Paradigalla* have a biofluorescent waddle, similar to males (figures 2 and 3). The biofluorescent plumage of females in *Diphyllodes*, *Paradisaea*, *Paradisornis* and *Semioptera* usually covers an analogous but smaller area than it does in males (figures 2 and 3). There was only one species, *Parotia berlepschi*, for which the AMNH does not have a female specimen. However, based on the examination of other females in *Parotia*, female *P. berlepschi* probably also exhibit biofluorescence on the mottled feathers of the chest and belly.

### 3.2. Biofluorescent emission spectra

**Figure 5.**
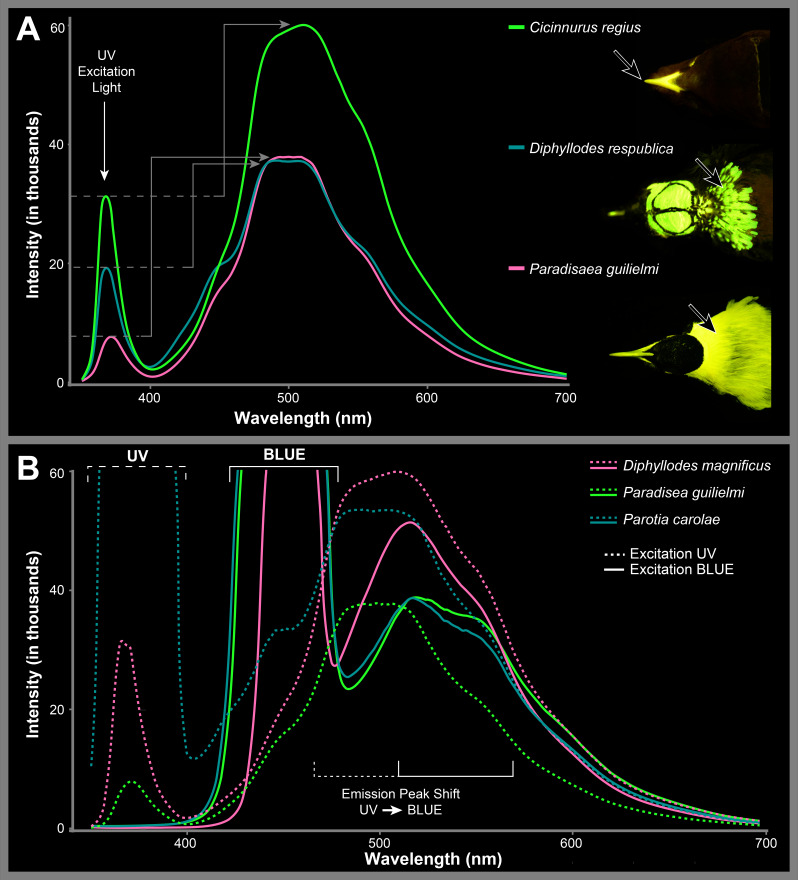
(A) Examples of spectral readings taken from biofluorescent patches excited by UV light showing how reflected UV excitation light can have a lower intensity than resulting biofluorescent emissions. Examples include the bill of *Cicinnurus regius* (AMNH 268198), neck plumage of *Diphyllodes magnificus* (AMNH 330409) and *Paradisaea guilielmi* (AMNH 679025). (B) Differences in fluorescent spectra from the same specimen under excitation via UV light and blue light.

Under blue excitation light, most species possess biofluorescent emission spectra that peak around 520 nm (green) and 560 nm (greenish-yellow; figure 4). If the peaks are unequal in intensity, the peak at 520 nm is usually more intense. Peak emission wavelengths are generally similar among biofluorescent patches on a single specimen and are the same across readings from multiple individuals of the sample species (example figure 4C). Under UV excitation light, these fluorescent spectra values are still emitted but with 560 nm not as a peak but as a shoulder value to a larger main peak between 470 and 520 nm (figure 5B). Excitation light in the UV range extends the emission spectra into a deep blue (shoulder emission values 445−455 nm in *Parotia carolae*), similar to the colour of our blue excitation light. This was the case for each of the five bird-of-paradise species tested under UV light (figure 5; table 1). In addition to a peak shift between UV and royal blue excitation light, we find that considerably less UV light intensity is reflected off the biofluorescent patches and the resulting biofluorescent emission spectra peaks have considerably higher intensity values (figure 5A). This is compared with royal blue excitation light, where excitation spectra reflected off specimens are considerably more intense than the fluorescent emissions they excite (figure 5).

Our resulting fluorescent emission readings are not a perfect indicator of the fluorescence intensity emitted by plumage, as these values are dependent on the intensity and orientation of the excitation light source as well as the distance the probe is to the structure being analysed. Although this is the case, the lower intensity values in females of most bird-of-paradise species do reflect the fact that, visually, these feathers (especially the mottled plumage on the belly) do not fluoresce as brightly as the patches found on males (electronic supplementary material, figure S3). Visualization of the fluorescence emission intensity differences can be seen in figures 2 and 3. Although most of the bright yellow and white plumage on birds-of-paradise was biofluorescent, emission spectra measurements of red/orange plumage (e.g. red plumes of male *Paradisaea*, orange patches on the head in *Parotia*, red plumage of *Cicinnurus*) and the bright blue and iridescent feathers (e.g. *Paradisornis* and *Ptiloris*) result in extremely low/zero fluorescence emission intensities (figure 4C; electronic supplementary material, figure S2B). This suggests that these plumages are not biofluorescent and do not convert UV or blue light into longer wavelengths. Lastly, all spectral measurements of black and iridescent plumage, common among many bird-of-paradise species, resulted in no fluorescent emissions (example measurement in figure 4C).

### 3.3. Biofluorescence in Ifritidae

Biofluorescence occurred in both male and female *Ifrita kowaldi*, the species belonging to the monotypic family Ifritidae and recovered as the sister group to the birds-of-paradise. The most striking biofluorescence occurred in the white eye stripe in males and the feet and bill of both sexes (electronic supplementary material, figure S4). Similar to birds-of-paradise, biofluorescent emission spectra peaked around 520 nm (green) and 560 nm (greenish-yellow) under royal blue excitation light. If the peaks are unequal in intensity, the peak at 520 nm is usually more intense (electronic supplementary material, figure S4).

### 3.4. Phylogenetic signal in biofluorescence

Using the bird-of-paradise phylogenetic tree from Ligon *et al*. [16] and adding *Ifrita kowaldi* as the outgroup, we performed stochastic character mapping of the presence or absence of biofluorescence to better understand the evolution of this trait across the group. Under both the equal-rates model and the symmetrical model, we find that the common ancestor of all birds-of-paradise probably possessed biofluorescence, which was subsequently lost in the ancestor of the clade comprising *Lycocorax*, *Manucodia* and *Phonygammus* (figure 1). Biofluorescence persisted in the rest of the lineage, considered the ‘core birds-of-paradise’. Based on the observations, we were able to make and spectra that could be recorded, we believe all species in this group also exhibit biofluorescence inside the mouth/throat in addition to the regions on the body as described above (figure 1).

## 4. Discussion

In this study we report, for the first time, the occurrence of biofluorescence in the birds-of-paradise. We find biofluorescence to be present in 37 of 45 bird-of-paradise species and 14 of 17 genera (all genera except *Lycocorax*, *Manucodia* and *Phonygammus*), representing all core birds-of-paradise (table 1; figure 1). Biofluorescence is a widespread phenomenon across the tree of life [30–33] and is hypothesized to be used in a variety of behaviours, including camouflage, prey capture and communication [43,46,58]. Very few studies have investigated biofluorescence in birds. Of those, roughly half have associated fluorescent emissions with courtship displays [26], sexual signalling [43,44] and sexual dimorphism [37]. Similar findings of sexually dimorphic biofluorescent patterns and signals have been reported in other organisms like sharks [31] and salamanders [32]. Additionally, the repeated evolution of biofluorescence and multiple occurrences of inter- and intraspecific variation in emission colour and patterning suggest its possible use in communication in fishes [30,59]. Although our findings add to the growing list of organisms potentially utilizing biofluorescence, the functional significance of these emissions remains largely under question. Birds-of-paradise are a charismatic group that use brightly coloured feathers in their visual displays [60,61], and we believe they may be using biofluorescence to enhance these signals during mating and arena presentations. Our reasoning is guided by the frameworks and criteria suggested by Marshall and Johnsen [46] and Nicolaï *et al*. [47] to determine whether biofluorescence may play a functional role in the birds-of-paradise. Based on their habitat, the location of their biofluorescent plumage and additional structures (e.g. inner mouths and waddles), their behaviour, and their visual capabilities, we argue that birds-of-paradise fulfil each prediction supporting the functional use of biofluorescence.

### 4.1. Excitation light and habitat use

The first criterion for visually significant biofluorescent visual cues requires that fluorophores be present in a location with enough excitation light for the signal emitter to use them [46]. Nicolaï *et al*. [47] and Taboada *et al*. [62] state that potentially favourable conditions in which biofluorescence may be used include not only aquatic environments but also terrestrial environments like shaded woodland habitats. With a few exceptions, most birds-of-paradise live as tree dwellers in tropical forests of New Guinea, the Moluccas and eastern Australia [63]. This equatorial location presents birds-of-paradise with consistent year-round solar irradiance. Most areas of Papua New Guinea receive an average of 6−7 kW m^−2^ d^−1^ (direct and indirect irradiation in September), categorized as ‘very high’ radiation [64]. Birds-of-paradise are forest dwelling and live in canopy areas. These habitats are complex and light spectra vary greatly within them, with radiance spectra in shaded forest canopies (heavy foliage) peaking between 500 and 650 nm (especially on sunny days) and with woodland shade (more treefall and thin crowns) having higher energy irradiance peaking between 300 and 475 nm [65]. Additionally, surface directionality of objects in forests is differentially affected by light in these areas [65]. For surfaces (including parts of organisms) facing upwards, light contribution from holes in the canopy is greater, whereas incident light affecting the side of a surface is more greatly affected by light filtering through vegetation [65]. All of these features of forested canopies affect visual signalling in birds-of-paradise.

As mentioned above, radiance spectra between 300 and 475 nm are higher in woodland shade habitats [65]. New Guinea, the Moluccas and eastern Australia are all home to both shaded forest canopies (spectra peaks approx. 500–650 nm) and shaded woodland habitats (spectra peaks approx. 300–475 nm). Different bird-of-paradise species display across the gambit of these light habitats. Some species display high up in the canopy (e.g. *Paradisaea*, *Seleucidis*) where full-spectrum sunlight can easily reach their plumage [49,66], whereas other species, like those in *Lophorina* and *Parotia*, display on the forest floor [16]. Males of these species choose sunny lek (mating grounds) arenas that are located where there are gaps in the canopy and where the light environment has a higher percentage of shorter (UV and blue) wavelengths [65]. Additionally, in many of the ground-displaying species, observing females perch above a displaying male, which better enables her to see a biofluorescent plumaged surface that is directed skyward and where light contribution from canopy holes has a greater effect [67]. Lastly, many species display in the understorey [16], which, depending on location, can have different spectral qualities. The spectral qualities of woodland habitats are of particular benefit to displaying birds-of-paradise, as these habitats contain a higher percentage of high energy (UV and blue) wavelengths, since bluish sky radiance dominates the irradiance spectrum [65].

We show that biofluorescent patches on birds-of-paradise absorb the high-energy ambient UV and blue light and re-emit it at longer wavelengths that are less common in woodland habitats (approx. 500–600 nm). Re-emittance at these wavelengths may increase the intensity of their already bright plumages, making them stand out in this more monochromatic environment. These are complex forest habitats, and spectral qualities can change drastically even 10 m into more densely forested areas. Regardless, birds-of-paradise live in geographic regions with an abundance of solar irradiance and their display behaviours suggest they may be preferentially utilizing different spectral qualities of the forest habitat in which they reside.

### 4.2. Plumage patterns, background and biofluorescent contrast

One problem organisms are likely to encounter when utilizing biofluorescence as a visual cue is signal loss of re-emitted light against complex and noisy backgrounds [46,47]. If biofluorescence functions as a signal, that signal can be enhanced when paired with contrasting borders or backgrounds [46]. This is observed in catsharks (Scyliorhinidae), where males and females possess sexually dimorphic fluorescent patterns. Bright green biofluorescence in catsharks increases contrast in body patterning at depth (greater than or equal to 30 m), making conspecifics easier to visualize in these low-light, essentially monochromatic oceanic environments [31,62]. Many bird-of-paradise species possess the epitome of contrasting plumage patterns and backgrounds. In *Paradisaea*, males possess brightly fluorescent heads, napes, bills and plumes; these regions are frequently bordered by starkly dark non-fluorescent iridescent green or dark brown plumages (figures 2, 4 and 5). A recent study shows that the black plumage of the core birds-of-paradise evolved to be ‘super black’, having increased structural light absorption compared with normal, moderately reflective, black feathers of other birds [68]. This ‘black hole’ effect is hypothesized to enhance the brilliance of adjacent colour patches, including the biofluorescent regions we observe in this study. Alternatively, the black plumage of *Lycocorax pyrrhopterus* (a bird-of-paradise species in the sister group to the core birds-of-paradise) does not possess ‘super black’ plumage, and was found in this study to not possess biofluorescence [68]. Across the lineage, all biofluorescent core birds-of-paradise possess some kind of contrast (electronic supplementary material, table S2; images). Whether it occurs as a fluorescent mouth surrounded by dark plumage, or as a gaudy patch used during a display, these contrasts are ubiquitous throughout the core birds-of-paradise. There are only eight species that possess these traits but in which we lack behavioural observations of the use of biofluorescent patches, *Astrapia nigra*, *A. rothschildi*, *A. splendidissima*, *A. stephaniae*, *Paradigalla brevicauda*, *P. carunculata*, *Parotia helenae* and *P. wahnesi*. Below we list some examples of biofluorescent contrasts exhibited by core birds-of-paradise.

Males and females of *Paradisornis rudolphi* have brightly fluorescent white-feather patches surrounding the orbit, and a highly fluorescent bill (figure 2). The contrast of these highly fluorescent regions is significantly increased as they are surrounded by super black head plumage. The fluorescent feathers on the nape of male *Diphyllodes magnificus* are fanned out and expanded during courtship displays and are bordered by dull-brown non-fluorescent plumage (figure 2). Additionally, bright blue skin on the head of *Diphyllodes respublica* is biofluorescent and contrasted by surrounding super black feathers. It is also criss-crossed by minute feathers in thin lines creating a sharply contrasting pattern (figure 5). The long white, fluorescent plumes of some male *Astrapia mayeri* are bordered by super black plumage (figures 2 and 4; electronic supplementary material, figure S1). The biofluorescent waddles of *Paradigalla* are surrounded by black/dark plumage and a non-fluorescent bill (figures 1 and 2), and male *Parotia* possess brightly fluorescent feathers near the nares, crown of the head and plumes, all of which are surrounded by super black plumage (figures 1, 2 and 4). *Seleucidis melanoleucus* possesses biofluorescent plumes covering its belly and flanks surrounded by super black and deep purple/green structural coloration on its wings (figures 1 and 2). Lastly, potentially all core bird-of-paradise species (figure 1) possess fluorescent regions inside their mouths (table 1; not imaged). Images in life show most of these bright neon green mouths bordered by darkly pigmented bills or feathers on the head, including species of *Drepanornis*, *Epimachus*, *Lophorina* and *Ptiloris*, which are either completely black or have dark structurally coloured plumage on the head.

In addition to contrasting plumages bordering or surrounding fluorescent feathers, 31 of the male paradisaeid species have been observed performing behaviours during courtship displays (electronic supplementary material, table S2) that increase contrast and may be enhanced by a biofluorescent signal [67,69–73]. Below we discuss a few of these displays; however, we also suggest the reader consult the copious number of studies describing these elaborate displays in detail [69–73]. Male *Drepanornis*, *Epimachus* and *Ptiloris* fan their shoulder plumes/wings up and around their face, creating a dark black halo surrounding their bill and displaying their biofluorescent mouth for extended periods of 30 s or more [69,70]. These behaviours performed during courtship displays greatly increase the amount of contrasting background around their open fluorescent mouths. Preceding a courtship display, male *Lophorina* perform a gape-open display towards females, flaring a black feathered cape while presenting an open fluorescent mouth [71]. Instead of displaying their fluorescent open mouth, male *Parotia* possess bright fluorescent patches of feathers on the nares or the crown of their head (figures 1, 2 and 4) and flare their black plumage out like a skirt [67,72]. To observing females sitting on branches overhead, this behaviour makes the biofluorescent feathers on their head strikingly contrasted against their super black skirt. *Parotia berlepschi* and *P. carolae* also possess white biofluorescent plumes next to their super black plumage (figures 1 and 4). These feathers are raised during courtship and become a brightly contrasted patch on their back once the super black ‘skirt’ is complete. Male *Pteridophora alberti* possess a light-coloured biofluorescent chest and belly (figure 1, electronic supplementary materials) and extremely elongate occipital plumes that alter the colour of reflected light (figure 1). During courtship, male *P. alberti* fluff themselves up, raise their black mantle cape and breast shield, and wave their fluorescent occipital plumes overhead, which are contrasted against their dark black cape [73].

Using the criteria outlined by Marshall and Johnsen [46] and Nicolaï *et al*. [47] for establishing a functional role for biofluorescence, we believe the enhanced contrast observed between biofluorescent body parts and the super black and dark plumages of many bird-of-paradise species provide compelling evidence for its use as an aid in visual signalling. Additionally, prior to courting, many paradiseaeid species perform behaviours that further increases fluorescent contrast with their surrounding backgrounds. Species that perform courtship displays near the ground, including *Diphyllodes*, *Lophorina* and *Parotia* often clear their court area (removal of leaves and twigs) prior to calling in females and performing their courtship displays [71,72,74]. Most behavioural studies agree that exposing the bare ground provides a plain backdrop that helps accentuate the colourful plumage of males in these species [72,74]. Whether or not the clearing of a court area is for the specific purpose of creating an enhanced visual signal is still unknown. Regardless, removing debris probably creates a less noisy background against which bright visual signals boosted by biofluorescence would appear enhanced.

### 4.3. Biofluorescent signal locations

In many biofluorescent organisms, the location of their fluorophores suggests that fluorescent emissions are either potentially utilized for camouflage [30] or are of no functional use [75]. The Marshall and Johnsen [46] and Nicolaï *et al*. [47] frameworks for determining whether an organism is using biofluorescence as a visual signal suggest fluorophores would be located on areas of the body that are specifically used in signalling behaviours. In the previous section, we provide evidence that birds-of-paradise utilize contrast and bright biofluorescence-enhanced plumage in courtship displays, which additionally offers support for the presence of fluorophores in areas of the body used for signalling. As previously mentioned, there are only eight species that possess biofluorescence and biofluorescent contrast but in which we lack behavioural observations of the use of fluorescent patches (electronic supplementary material, table S2). Here, we give examples of additional species that utilize expansive and flashy areas of plumage as visual cues during courtship and hierarchical displays, which may be enhanced by their biofluorescent properties, focusing on three monotypic genera (*Cicinnurus*, *Seleucidis* and *Semioptera*) and one diverse genus (*Paradisaea*) that display in this manner.

In the monotypic genus *Seleucidis* (*S. melanoleucus*), males are fluorescent over the entire lower half of their body, including the belly feathers, rump, plumes and legs (figures 1 and 2). They possess a striking contrasting border between their upper body plumage, which is a combination of stark super black and darkly iridescent feathers, and their biofluorescent lower plumages (similar to the discussion above focusing on contrasting borders). Prior to courting, male *S. melanoleucus* open their bright green fluorescent mouths while calling towards distant females. During courtship, male *S. melanoleucus* raise their dark wings and present their fully exposed brightly fluorescent flank plumes and thighs to a female [66]. Males of the monotypic genus *Cicinnurus* (*C. regius*) possess brightly fluorescent plumage on the flank, belly and chest (figures 1 and 2) and also perform a rump display towards females during courtship [60]. Compared with the intensely biofluorescent emissions from the plumage of other birds-of-paradise, the monotypic genus *Semioptera* (*S. wallacii*) is comparatively dull under both white light and blue excitation light and lacks stark contrasting plumages (figure 1; electronic supplementary materials). Males of *S. wallacii* possess brightly fluorescent legs, moderately fluorescent plumes and fluorescent trailing ends to their primaries (figure 1). During displays, males flair out and aim their plumes towards a female, raising their wings over and behind their heads to create a fluorescent halo-like effect [76]. Thus, the males in each of these monotypic genera are utilizing plumages during courtship displays, and the additional presence of biofluorescence may be enhancing these visual signals. In these instances, we find that fluorescent plumages used for displays vary in shape, colour and size among male birds-of-paradise (figure 2). In more species-rich genera like *Paradisaea*, biofluorescent plumage generally occurs in similar locations among species within the genus (figure 4*b*) and is similarly used in large, bright, flashy courtship displays.

*Paradisaea* is one of the most speciose and well-studied of all the bird-of-paradise genera (six species) [49]. In this genus, fluorescent plumage of males covers the majority of the head, neck and back (figures 2 and 4), extending onto their large and extravagant plumes (figure 1; electronic supplementary material, figure S1). These fluorescent regions are usually bordered and contrasted by smaller patches of non-fluorescent structurally green or super black plumage on the head or neck (figure 2). Males additionally have large areas of non-fluorescent brown plumage on the wings, chest, back and belly (figure 2; electronic supplementary material, figure S1). Male *Paradisaea* greatly utilize their bright heads and plumes in a variety of courtship and male hierarchical displays, often conducted in groups. In many of these displays, including the ‘wing pose’ and the ‘charging display,’ males raise and spread their bright plumes while hopping and moving through a spasm of postures [49]. In the ‘charging display’, ‘zigzagging’ and ‘flower display’, males bow down their bright fluorescent head and bill in a position that creates significant contrast against their darker non-fluorescent plumage [49]. The ‘inverted display’, believed to be common among all *Paradisaea* species, greatly emphasizes bright fluorescent plumage [49]. Males hang upside-down from a perch, extend their neck, flair their plumes, and often begin swaying or rotating, making their bright fluorescent plumes even more conspicuous [49]. These myriad postures and displays in *Paradisaea*, and throughout all core birds-of-paradise, utilize brightly coloured plumage as a visual signal. Biofluorescence is a common component of these plumages and may function to make these signals even more eye-catching during courtship and hierarchy displays by enhancing brightness and intensity.

### 4.4. Bird vision and spectral sensitivities

Visual cues and signals can only serve a functional role if they are perceived by the intended signal-receivers. Marshall and Johnsen [46] predict that if biofluorescent emissions function as a signal, receiving individuals should have optimal spectral sensitivities to visualize the emitted wavelengths. Birds possess greater visual acuity and spectral awareness relative to many other vertebrates, as they have small eyes which have been found to correlate with increased acuity and possess additional photoreceptor cones, allowing many species to visualize spectra in the ultraviolet sensitive (UVS) and violet sensitive (VS) wavelength ranges [23,25–27,77,78]. Birds also possess light-filtering oil droplets of different colours (clear, red, yellow) on each photoreceptor cone [25,28]. Wavelengths that reach the cone receptor are narrower in bandwidth than they would be without these oil droplets. Thus, the increased spectral sensitivity of each cone type allows birds to better discriminate between colours. Pigmented oil droplets are also believed to act as longpass cut-off filters, blocking out shorter wavelengths [79].

Birds-of-paradise are inferred to have spectral sensitivities in the VS spectrum, with VS cone type peaks at 402−426 nm and with additional cone peaks at approximately 480 nm (short-wavelength sensitive (SWS) cone type), 540 nm (middle-wavelength sensitive (MWS) cone type) and approximately 610 nm (long-wavelength sensitive (LWS) cone type) [16,77,78]. This is unlike many UVS sensitive bird species that can visualize light peaks in the UV range around 355−380 nm [78]. We find that excitation via UV light (355–380 nm) results in intense biofluorescence in birds-of-paradise peaking around 480−530 nm (figure 5), which is a close match between the spectral sensitivity in the SWS and MWS cones in VS species like birds-of-paradise. The intensity in the fluorescent peaks even exceeds that of the UV excitation light, adding perceptible volume to their visual space (figure 5A). Birds-of-paradise are enhancing brightness of already flashy plumage by converting high-energy light from the UV and blue portions of the spectrum, where their visual sensitivity is low, and re-emitting it at wavelengths where their visual sensitivity is considerably higher.

Birds-of-paradise have evolved contrasting plumages and perform elaborate behaviours that utilize visual signals [46,80]. These signal locations consistently include fluorescent plumage that may function to increase the brightness of these regions, which would enhance contrast between bright plumage and body regions used as visual signals that are bordered or surrounded by non-fluorescent dark/super black plumage. The presence of pigmented oil droplets on the photoreceptor cones of the eyes in birds-of-paradise may aid visualization of fluorescent emissions, making these signals even more pronounced. Whether by increasing the sensitivity of each cone-type or by acting as a longpass filter, filters in the eye may allow for better visualization of longer wavelength biofluorescent light by blocking higher energy, shorter wavelengths (UV, violet, blue), thereby enhancing contrast [81].

### 4.5. Biofluorescence in female birds-of-paradise

Unlike males, females do not perform elaborate courtship or hierarchical displays, and the location of their biofluorescent plumage is much more restricted. In females, biofluorescence is generally limited to the lighter portion of the mottled feathers on the ventral surface of their bodies. An exception to this occurs in *Paradigalla*, *Paradisaea* and *Paradisornis*, where females instead exhibit similar, but less expansive, patterns as males (figure 3). Currently, our findings do not offer compelling evidence that female birds-of-paradise exploit biofluorescence to enhance visual signalling. It is possible, especially in females with similar biofluorescent regions and patterns to males, that it is a by-product of its evolution and possible positive selection in males. This pattern only occurs in females of eight (five of which are in *Paradisaea*) of the 45 species of birds-of-paradise. Alternatively, in the females of the remaining 37 species, biofluorescence is generally restricted to the mottled feathers on the ventral surface. Fluorescent plumage of these species is possibly being used for other purposes such as camouflage. Biofluorescence is hypothesized to be used for camouflage in many organisms [82,83]. When used in this way we often find that an organism’s fluorescent emissions match its background or aid in enhancing a ‘noisy’ visual pattern that blends in well with complex, busy backgrounds. The mottled/scaled feathers and drab plumage of many female bird species are a type of static camouflage believed to aid in background matching and disrupting the silhouette [84,85]. Most female bird-of-paradise species possess this type of plumage patterning (figure 3), suggesting their plumage is, likewise, important for camouflage. The fluorescent properties of the lighter portion of their mottled feathers might enhance their ability to disrupt their silhouette while in the canopy and/or foliage. Alternatively, many of these females also possess fluorescent eyebars (e.g. figure 3, female *Diphyllodes magnificus*), which could aid as a visual signal for males that an observing female is watching a mating display. Although further study is needed, the presence of a variety of patterns on the body of female birds-of-paradise may allow for simultaneous camouflage and communication [85].

## 5. Conclusions

In this study, we show for the first time that birds-of-paradise (Paradisaeidae) are biofluorescent, and that this phenomenon is present in all core bird-of-paradise species (figure 1; table 1). Using established frameworks and criteria for determining whether fluorescence serves a functional role [46,47], we investigate whether there is compelling evidence that biofluorescence functions as a signal during courtship and mating displays in birds-of-paradise. We found that most of the bright yellow and white plumage on birds-of-paradise is biofluorescent. The presence of white or light-coloured feathers (i.e. that may contain fluorophores that result in fluorescence) in the majority of avian lineages indicates that fluorescence may be far more widespread in birds than what has been reported in the literature to date. The current very limited number of studies reporting fluorescence in birds [34–45] suggests this phenomenon has not been thoroughly investigated across their radiation and highlights the need for more taxonomically diverse studies.

Birds-of-paradise are a charismatic group in which males are well known for their elaborate feather development and bright coloration. Their unique mating rituals and displays have fascinated scientists and spurred a myriad of studies focused on trait evolution and sexual selection [18–20]. After analysing museum specimen skins of all 45 currently described bird-of-paradise species, we find that 37 species and 14 of the 17 genera are biofluorescent, including all core birds-of-paradise (all genera except *Lycocorax*, *Manucodia* and *Phonygammus*) (figure 1; table 1). Under blue light, the primary wavelengths emitted from biofluorescent feathers and other regions of the body are within the green and yellow regions of the spectrum, with most species emitting two colour peaks from a single fluorescent patch: one around 520 nm and another around 560 nm (figure 4).

Birds-of-paradise occur across eastern Australia, Indonesia and New Guinea near the equator, where there is an abundance of bright solar light year-round that can readily excite fluorophores [54]. They live in forests where the complexity, filtering and spectra of light are significantly affected by differences in canopy density and where biofluorescent signals may be enhanced [65]. In males, biofluorescence occurs on bright plumage and skin that are highlighted and used during arena and courtship displays, hierarchical structuring or prior to copulation with a female. These locations vary among species but include the inner mouth and bill, feathers on the head and neck, feet and plumes (figures 1, 2 and 4). In most cases, whether by presence of contrasting plumage or via behaviour of the individual, biofluorescent areas of the body are starkly contrasted and stand out against the surrounding environment, particularly during male display rituals [60,61,71,72,74,86]. Although no studies have specifically assessed vision in birds-of-paradise, character reconstructions of their visual capabilities through phylogenetic inference suggest males and females are probably only violet sensitive, and unable to visualize UV light [16,77,78]. Instead, spectral peaks for two of the cones in their eyes match those of their biofluorescent emissions (480–530 nm). Additionally, various traits within the eyes of birds (like an extra photoreceptor cone and pigmented oil droplets) allow them to visualize light in additional ways than many other vertebrates [16,25,28,78].

Based on their habitat, the location of their biofluorescent plumage and additional fluorescent structures (e.g. inner mouths and waddles), their behaviour, and their visual capabilities, birds-of-paradise fulfil each of the criteria outlined by Marshall and Johnsen [46] and Nicolaï *et al*. [47] for attributing function to biofluorescence and that can be used to determine whether biofluorescent emissions should be considered visually significant to the individuals expressing them. Specifically, we show that for birds-of-paradise: (i) fluorescent molecules (fluorophores) can take advantage of excitation light wavelengths in the habitat they live in; (ii) fluorescent signals are viewed next to or against contrasting backgrounds; (iii) fluorescent signals are placed on areas of the body that are used in signalling behaviours; and (iv) that signal-receivers have optimal spectral sensitivities to the emitted wavelengths. Our results provide compelling evidence that within the core birds-of-paradise, males are utilizing biofluorescence to enhance already bright visual cues used during courtship and arena displays, which may aid in male competition and copulatory success. This research adds to the growing list of avian lineages that have been shown to exhibit biofluorescence.

## Data Availability

Data are included as supplementary material [87].
